# Development of an Inhibition Enzyme-Linked Immunosorbent Assay (ELISA) Prototype for Detecting Cytotoxic Three-Finger Toxins (3FTxs) in African Spitting Cobra Venoms

**DOI:** 10.3390/molecules27030888

**Published:** 2022-01-28

**Authors:** Ernest Z. Manson, Kyama C. Mutinda, Joseph K. Gikunju, Aleksandra Bocian, Konrad K. Hus, Vladimír Petrílla, Jaroslav Legáth, James H. Kimotho

**Affiliations:** 1 Institute for Basic Sciences, Technology & Innovation, Pan African University, Nairobi 00100, Kenya; 2 Department of Medical Laboratory Science, College of Health Sciences, Jomo Kenyatta University of Agriculture & Technology, Nairobi 00100, Kenya; mkyama@jkuat.ac.ke (K.C.M.); jgikunju@jkuat.ac.ke (J.K.G.); 3 Department of Biotechnology and Bioinformatics, Faculty of Chemistry, Rzeszow University of Technology, 35-959 Rzeszow, Poland; bocian@prz.edu.pl (A.B.); k.hus@prz.edu.pl (K.K.H.); jaroslav.legath@uvlf.sk (J.L.); 4 Department of Biology and Physiology, University of Veterinary Medicine and Pharmacy, 041 81 Košice, Slovakia; vladimir.petrilla@uvlf.sk; 5 Zoological Department, Zoological Garden Košice, Široká 31, 040 06 Košice-Kavečany, Slovakia; 6 Department of Pharmacology and Toxicology, University of Veterinary Medicine and Pharmacy, 041 81 Košice, Slovakia; 7 Kenya Medical Research Institute, Nairobi 00100, Kenya; jhkimotho@kemri.org

**Keywords:** inhibition ELISA, three-finger toxins, cytotoxic, snakebite envenoming, diagnostics

## Abstract

The administration of toxin-specific therapy in snake envenoming is predicated on improved diagnostic techniques capable of detecting specific venom toxins. Various serological tests have been used in detecting snakebite envenoming. Comparatively, enzyme-linked immunosorbent assay (ELISA) has been shown to offer a wider practical application. We report an inhibition ELISA for detecting three-finger toxin (3FTx) proteins in venoms of African spitting cobras. The optimized assay detected 3FTxs in *N. ashei* (including other *Naja* sp.) venoms, spiked samples, and venom-challenged mice samples. In venoms of *Naja* sp., the assay showed inhibition, implying the detection of 3FTxs, but showed little or no inhibition in non-*Naja* sp. In mice-spiked samples, one-way ANOVA results showed that the observed inhibition was not statistically significant between spiked samples and negative control (*p*-value = 0.164). Similarly, the observed differences in inhibition between venom-challenged and negative control samples were not statistically significant (*p*-value = 0.9109). At an LOD of 0.01 µg/mL, the assay was able to confirm the presence of 3FTxs in the samples. Our results show a proof of concept for the use of an inhibition ELISA model as a tool for detecting 3FTxs in the venoms of African spitting cobra snakes.

## 1. Introduction

Snakebite envenoming constitutes a major public health challenge in developing countries. It contributes significantly to cases of morbidity and mortality, particularly in poorly developed regions including sub-Saharan Africa and Asia [1]. The World Health Organization estimates that about 5.4 million people globally are bitten by snakes every year [2]. Of this, nearly 2.7 million people are believed to be envenomed, resulting in between 81,000 to 138,000 deaths [1]. Although likely grossly underreported, close to 15,000 snakebites occur each year in Kenya [3]. The African spitting cobra species comprises of various snake species including *Naja nigricollis*, *Naja mossambica*, *Naja katiensis*, *Naja pallida*, and others. *Naja ashei* belongs to the African spitting cobras, which are distributed widely across sub-Saharan Africa. *N. ashei*, also called the large brown spitting cobra was in 2007 considered a distinct species and classified accordingly [4]. As is the case with the other African spitting cobras, the venom of *N. ashei* is known to induce mainly cytotoxic effects caused by the predominant presence of 3FTx proteins and phospholipases A_2_ [5,6]. *N. ashei* is a medically important snake species in Kenya, Ethiopia, Uganda, Somalia, and Tanzania, and thus, is implicated in snakebites and associated morbidity, mortality, and disability [7].

Proteomic analysis of the venom of spitting cobras shows that the venoms are made up largely of three-finger toxins (3FTxs), accounting for about 67–80%. For instance, 3FTx proteins were found to constitute 73.3% of the total venom load of *N. nigricollis* [8]. Similarly, an analysis of the venom proteome of *N. ashei* revealed that 3FTx proteins accounted for 60–80% [5,6]. Three-finger toxins are non-enzyme-based polypeptides present in the venoms of elapids and colubrids [9,10], and are very rare in the venoms of vipers [11]. Three-finger toxins present in some elapid venoms are cytotoxic and are thus capable of inducing tissue necrosis [9]. They also include cytolytic cardiotoxins, which are mainly involved in the formation of ion pores [12].

Among other things, improved diagnostic methods are crucial in discriminating between various venoms [13]. With advancements in diagnostics capable of detecting specific toxins, implicating species in snakebite envenoming could potentially be deduced, allowing the administration of toxin-specific treatments as well as other species-specific bites [12,14]. Various serological tests are available for detecting snake venoms including agglutination assays, enzyme-linked immunosorbent assays (ELISA), florescence immunoassay, and others [15]. However, ELISA has been shown to offer superior practical application relative to the other tests [15,16,17] and has been employed clinically in the diagnosis of snakebites and studying envenoming-associated syndromes [15,16,17,18,19]. Inspite of this, ELISAs are not readily deployed in the field owing to constraints of time and requirements for specialized equipment and reagents [17,20,21], albeit the technique is currently being used for clinical purposes [22]. Even more promising is the fact that ELISA is the basis for developing snake venom detection kits, such as the CSL-SVDK, which is considered the only commercially available device for detecting snakebite envenoming [23]. In this study, we describe an inhibition ELISA for detecting 3FTx proteins, the most abundant toxin present in the venoms of African spitting cobras. The assay was tested for its ability to discriminate 3FTxs-containing and non-3FTxs-containing venoms. An inhibition ELISA is a variant of ELISA where an antigen-containing sample and primary antibody are incubated for specific binding. Following the incubation process, the resulting antigen-antibody complex is transferred to a plate coated with a standard antigen, resulting in binding between the coated antigen and free primary antibody. Due to the initial binding between the antigen-containing sample and primary antibody, there is a reduction in the reaction (reduced absorbance), which is inversely proportional to the analyte concentration in the sample [24].

An inhibition ELISA assay was developed, optimized, and evaluated for the detection of 3FTx proteins in African spitting cobras. The assay was able to detect 3FTxs in both spiked samples and venom-challenged mice samples.

## 2. Results

### 2.1. Optimization of ELISA Parameters

Following optimization of ELISA parameters, the highest signal-noise ratio of the conjugate was obtained at a dilution of 1:8000. Also, significant levels of detection were attained at 1 µg/mL of the coating antigen concentration. A two-tailed t-test analysis of substrate sensitivity showed that OPD produced significantly higher ODs compared to TMB (*p*-value = 0.0207). Consequently, these parameters were considered optimal and thus adopted for subsequent assays.

### 2.2. Determination of ELISA Cut-Off Point

The specificity of the assay was evaluated using homologous (same genera) and heterologous (different genera) venom samples. At all the inhibitor concentrations tested, a percent inhibition of greater than 18% was observed across all homologous venoms, with the pattern more closely related between *N. ashei* and *N. nigricollis* (18.80 and 21.00% respectively). On the other hand, a% inhibition less than 16% was observed among the heterologous samples across all the inhibitor concentrations tested (Table 1). On the basis of the specificity of the assay for both homologous and heterologous samples, a percent inhibition of greater than 30% was set as the identification criteria for the presence of the analyte of interest in homologous venom samples [24].

Sensitivity of the inhibition ELISA was also determined using crude *N. ashei* venom at various concentrations. Negative controls made up of pre-immune mice serum samples were also analyzed. With a mean and standard deviation (of the negative control) of 0.835 and 0.207 respectively, the limit of detection (LOD) of the assay was determined to be approximately 0.01 µg/mL (Table 2). Although the lowest concentration in the assay was 0.04 µg/mL, the determined OD was 1.249 which fell between 0.04 µg/mL and the ‘No Antigen Control’ (NAC), hence the 0.01 µg/mL. This implies that at a minimum concentration of 0.01 µg/mL, the inhibition ELISA assay was capable of discriminating between positive and negative samples. Similarly, at the same cut-off, the assay was able to confirm the presence or otherwise of 3FTxs in a venom sample. It should be noted that familiarity with inhibition ELISA coupled with ‘NAC’ sample type may give rise to variations in cut-offs. Nevertheless, ODs of positive and negative control samples should be consistently different. As happens with inhibition ELISAs, lower ODs imply high levels of the analyte of interest, thus more competitive binding and reduced signal (OD) and the vice versa. 

### 2.3. Evaluation of the Developed Prototype

#### 2.3.1. Inhibition ELISA for Detecting Three-Finger Toxins in Crude *N. ashei* and Other Venoms

**Figure 1:**
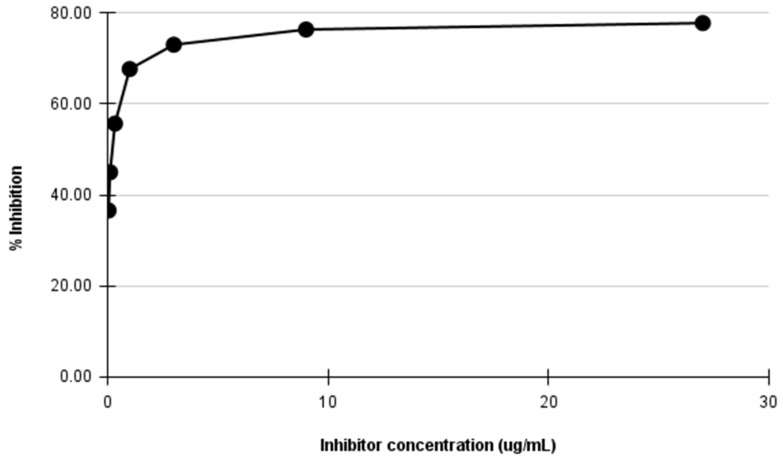
Inhibition ELISA curve showing % inhibition of the coated antigen by the sample-containing antigen at various concentrations of crude *N. ashei* venom (inhibitor). Sample antigen was diluted 3-fold from an initial concentration of 27 µg/mL to a final concentration of 0.04 µg/mL and run in duplicates.

From the six-point three-fold dilution of the sample antigen, the percent inhibition was observed to be highest (77.70%) at 27.00 µg/mL and lowest (36.56%) at 0.04 µg/mL of the antigen, respectively (Figure 1). In terms of absorbance, the ODs at these two concentrations of the sample antigen were 0.380 and 1.081, respectively. In contrast, the ‘No Antigen Control’ recorded an OD of 1.704 (Table 3). The results indicate that the 3FTxs present in the crude *N. ashei* venom were inhibited across all the concentrations tested when compared with the NAC. The inhibition is reflected in the reduced signals (ODs) particularly in wells containing high concentration of the antigen. Suffice to say, in these wells, there were more 3FTxs antigen, resulting in more binding between the primary antibody and the antigen, hence the high percent inhibition. 

The results of the inhibition ELISA were used to confirm, first of all, the presence of three-finger toxins in the crude venoms assayed and secondly, to determine the % inhibition caused by the different sample antigens present in the crude venoms at the different concentrations tested. The ANOVA summary (Table 4) shows that there was a significant difference (*p*-value < 0.0001) within the sample antigen-induced inhibition in terms of the means. Nearly 72% of the variation in inhibition caused by the sample antigen therein in the crude venoms is explained by the analysis (R-squared = 0.7185).

From the post hoc results (Table 5), it can be observed that there was a significant difference in the inhibition caused by the 3FTx-containing sample antigens between all three *Naja* species and their non-*Naja* counterparts, with the difference being particularly significant between *N. nigricollis* vs. *B. arietans*, *N. nigricollis* vs. *D. polylepsis*, *N. haje* vs. *B. arietans*, and *N. haje* vs. *D. polylepsis* (*p*-value < 0.0001 in all cases). Also, there was no significant difference in the 3FTx-induced inhibition between all three spitting cobra species. The multiple comparisons test also show that the sample antigens-induced inhibition (if any) between *B. arietans* vs. *D. polylepsis* was not significant (*p*-value = 0.9999).

**Figure 2:**
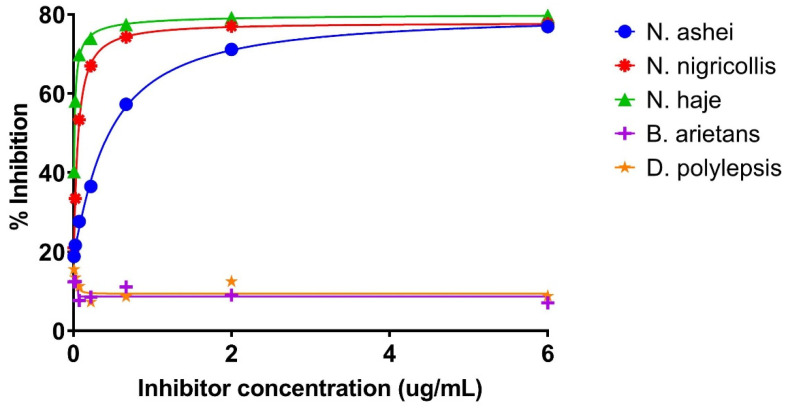
Inhibition ELISA curves showing % inhibition of the coated antigen (pure 3FTx fraction) by sample antigens from crude *N. ashei*, *N. nigricollis*, *N. haje*, *B. arietans*, and *D. polylepsis* venoms at different concentrations. The different sample antigen-containing venoms were diluted 3-fold from an initial concentration of 6 µg/mL to a final concentration of 0.008 µg/mL. Samples were then analyzed in duplicates at each concentration.

It was also be observed that at high inhibitor concentrations (Figure 2), the ODs of all three *Naja* species were low. The ODs, however, increased with decreasing inhibitor concentrations. As low as 0.008 µg/mL inhibitor concentration, the sample antigens induced varying levels of inhibition (18.80, 21.00, and 40.28% for *N. ashei*, *N. nigricollis*, and *N. haje*, respectively). Conversely, increasing inhibitor concentrations resulted in an increase in the percent inhibition for the same species.

For all the venoms tested, the ‘No Antigen Control’ (NAC) produced the highest OD values, an indication of inhibition when compared with wells containing different inhibitor concentrations. ODs obtained for *B. arietans* and *D. polylepsis* suggest that there was no or minimal inhibition mainly because the venoms either contain little or none of the antigen of interest. As such, the ODs are comparable to those of the NAC (Table 1). 

#### 2.3.2. Inhibition ELISA for Detecting 3FTx Proteins in Spiked Samples

**Figure 3:**
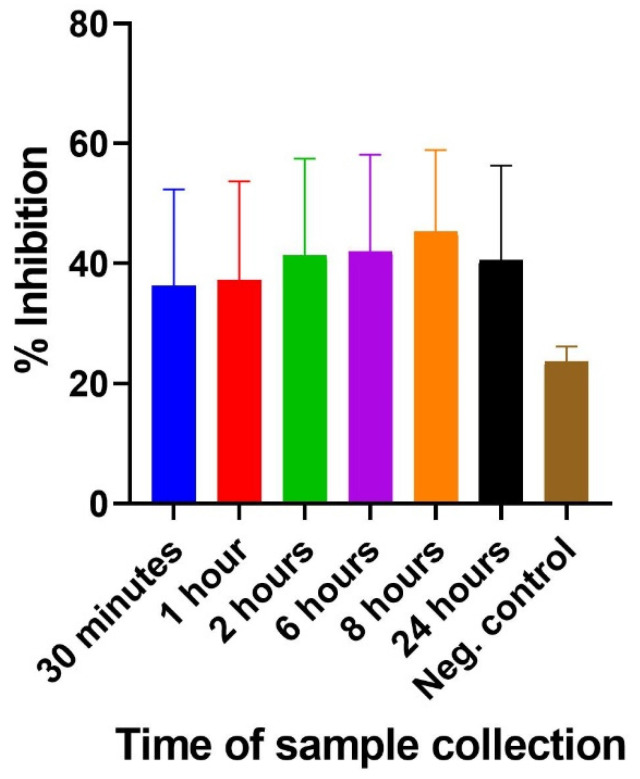
Comparison of % inhibition of coated antigen (3FTxs) by *N. ashei* venom spiked samples collected at different times (30 min–24 h).

The utility of the inhibition ELISA was further tested for its capacity to detect 3FTxs in blood samples spiked with crude venom at different times. The spiked samples were assayed alongside previously collected pre-immune serum samples as control. An analysis of the results shows that there was inhibition of the coated antigen (purified 3FTxs) by the *N. ashei* 3FTxs-containing venom at all the dilution points. In contrast, the negative control showed no inhibition. The % inhibition was largely similar across the different sample collection times, but varied significantly relative to the negative control. One-way ANOVA results showed no significant difference among the means (*p*-value = 0.164) in terms of sample collection times. Similarly, no significant difference was found between the negative control and the other samples collected at the respective times. The results nonetheless demonstrate that the inhibition ELISA is capable of detecting 3FTxs in blood samples spiked with crude venom as depicted in the Figure 3 below.

#### 2.3.3. Inhibition ELISA for Detecting 3FTxs in Mice Challenged with *N. ashei* Venom

**Figure 4:**
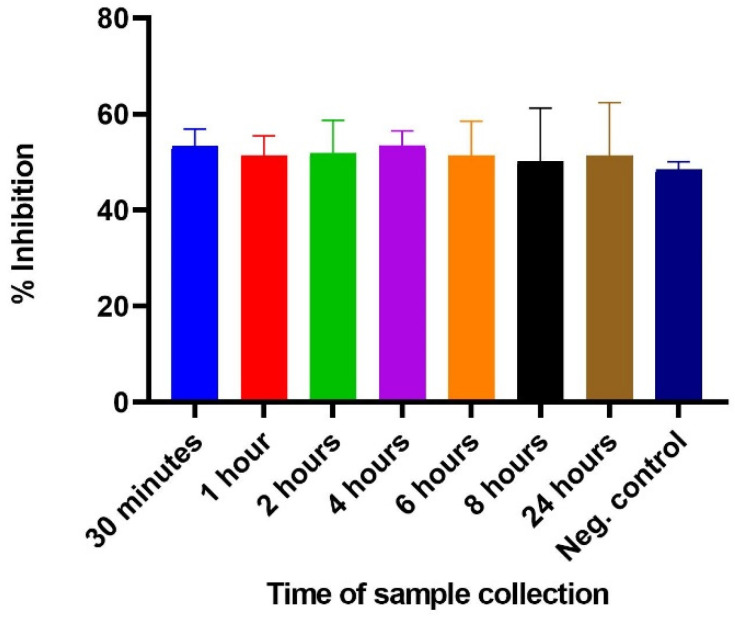
Comparison of % inhibition of coated antigen (3FTxs) by *N. ashei* venom in challenged mice with samples collected at different times (30 min–24 h).

The potential use of the inhibition ELISA in snakebite envenoming was evaluated in mice challenged with crude *N. ashei* venom. The results demonstrate that 3FTxs were detected, evidenced in the observed inhibition in the sample-containing wells. Albeit varied inhibition between the samples and negative control were observed, the differences were not statistically significant (*p*-value = 0.9109) as determined by one-way ANOVA. Similarly, multiple comparison analysis showed that the inhibition between the negative control and the samples collected at different times were not significantly different. It is worthy to note that regardless of the differences, the assay was able to detect 3FTxs in the samples tested as shown in the observed inhibition (Figure 4). 

## 3. Discussion

The determination of optimal concentrations for antigen coating, dilutions of primary and detection antibodies, and substrate sensitivity among others is crucial for the development of a successful assay [24]. In line with this, antigen coating concentration of 1 µg/mL was found to be optimal as previously reported by Laustsen and colleagues [25]. A 1:8000 dilution of the detection antibody was found to be more beneficial and represents a more cost-effective choice. Also, OPD, in addition to showing consistently higher absorbance readings was found to be statistically different from TMB, and thus, adopted for subsequent assays albeit Hosoda et al [26] and Goka and Farthing [27] had previously reported contradictory observations. 

The results of the inhibition ELISA indicate that the 3FTxs present in the crude *N. ashei* venom were inhibited across all the concentrations tested when compared with the NAC. The inhibition is reflected in the reduced signals (ODs), particularly in samples containing high concentrations of the antigen. Suffice to say, in these wells, there were more 3FTxs antigen, resulting in more binding between the primary antibody and the 3FTxs antigen, hence the high percent inhibition. ODs obtained for *B. arietans* and *D. polylepsis* suggest that there was no or minimal inhibition mainly because the venoms either contain little or none of the antigen of interest. As such, the ODs are comparable to those of the NAC (Table 1). The case of *B. arietans* could be attributed to the fact that 3FTxs are thought to be uncommon in viper venoms [11]. *D. polylepsis*, although an elapid too, is reported to have 31.0% of its total protein content made up of 3FTxs toxins [28]. However, a majority of these toxins are neurotoxic (including muscarinic and short neurotoxins) and appear to lack the cytotoxic 3FTxs that characterize the *Naja* sp., hence the seeming absence of inhibition as observed earlier. Again, the preponderance of neurotoxic 3FTxs in the venom proteome of *D. polylepsis* is supported by Blanchet et al. [29]. Thus, the apparent lack of inhibition as observed in *D. polylepsis* may be attributed to the high level of neurotoxic 3FTx proteins as previously reported [28,29]. The relatively high and comparable inhibition observed in *N. ashei*, *N. nigricollis*, and *N. haje* may confirm two things; first, the similarity in the venom proteomes of the cobras (both spitting and non-spitting) and second, the venom proteomes of *Naja* sp. contain more 3FTxs. This finding is consistent with Hus et al. [5,6] and Petras et al. [8], who found that proteomic analysis of spitting cobra venoms shows that 3FTxs proteins make up the bulk. Hus and colleagues [5,6] found that 3FTxs accounted for 60–80% of the protein composition of *N. ashei* venom, while the same toxin was found to constitute 67–73% of *N. nigricollis* venom [8]. Thus, the inhibition patterns demonstrate that all three *Naja* species are made up of largely 3FTxs. Also, the similarities in the inhibition among the three *Naja* snakes is supported by Casassola et al. [30] who observed that similar biochemical, antigenic and toxinological characteristics are shared by snake venoms that belong to the same genera. Similarly, the venom of *Hemachatus haemachatus*, an African spitting cobra-like species found predominantly in Southern Africa was reported to contain 63.3% 3FTx proteins [31], although it is closely related to the *Naja* genus phylogenetically.

A variety of methods are available for determining the analytical sensitivity of assays [32,33,34]. The determination of limit of detection of an assay among other parameters is required for establishing limits of identification. From the percent inhibition of the sensitivity assay, the LOD was determined to be 0.01 µg/mL. At this cut-off, the assay was able to confirm the presence of the analyte of interest in the samples tested. The detection of envenoming by our inhibition ELISA model is consistent with previously reported ELISA methods, although different LODs were found. An avidin-biotin ELISA kit by Dong and colleagues reported a detection limit of 0.2–1.6 ng/mL in venom samples from South Vietnam [17]. In aiding the management of snakebites in Taiwan, a sandwich ELISA approach was found to have a limit of quantification of 0.78 and 0.39 ng/mL for haemorrhagic and neurotoxic venoms, respectively [35]. Similarly, Shaikh and colleagues, in developing a sandwich ELISA assay for detecting venoms of common Indian snakes found the sensitivity of the assay to be 0.01 ng/mL [36]. Additionally, Watanabe et al. [37] and Selvanayagam [38] in their respective ELISAs for detecting snake venoms and toxins in mice serum and tissue homogenate, respectively, found sensitivities of 50 and 0.1 ng/mL. These studies, when considered in combination with our findings demonstrate capacity and utility of various ELISA methods to detect snake venom in different samples. Thus, albeit rarely used in snakebite envenoming, inhibition ELISA could prove useful in detecting cobra venoms.

In both blood samples spiked with crude venom and mice challenged with the same, the inhibition ELISA was able to detect the presence of 3FTx proteins, as demonstrated in the observed differences in % inhibition when compared to the negative control. This finding is significant to the extent that such an assay could be useful not only in discriminating between venoms [13], but also allow the identification of snakes implicated in envenoming and ultimately pave the way for the administration of toxin-specific therapies [12,14]. Thus, although preliminary, the inhibition ELISA may prove useful in snakebite envenoming, although it is rarely employed in this field.

## 4. Materials and Methods

### 4.1. Snake Venoms

Lyophilized crude *N. ashei* venom was obtained from snakes maintained at the Bioken snake farm in Watamu, Kenya. The venom was reconstituted in distilled water and stored at −20 °C until use. Three-finger toxin (3FTx) fraction was purified using size-exclusion chromatography as described elsewhere [39]. Venoms from *Naja nigricollis*, *Naja haje*, *Bitis arietans*, and *Dendroaspis polylepis* were obtained from Baringo Snake Park, Kenya, in liquid form.

### 4.2. Animals

Eight-week-old adult female Balb/c mice were challenged with 3FTxs (50 and 100 µg). Animals were sampled in February 2021 fortnightly in the tail vein for antibody screening for about 14 weeks. Blood samples were centrifuged to obtain serum, which were then stored at −20 °C until use, which antibodies were used as primary antibodies in all experiments. Animals were kept at the animal facility of the Kenya Medical Research Institute (KEMRI) and provided with food and water *ad libitum*.

### 4.3. Ethical and Institutional Approvals

Approval for the study was obtained from the KEMRI Center for Biotechnology Research and Development (CBRD), KEMRI’s Animal Care and Use Committee (ACUC), and KEMRI Scientific and Ethical Committee Unit (SERU) with protocol number KEMRI/SERU/CBRD/229/4340.

### 4.4. Quantification of Protein Content of Venoms

Protein content of all venoms was determined using the BCA kit in line with the manufacturer recommendations [40].

### 4.5. Optimization of ELISA Parameters

Goat anti-mouse HRP conjugated antibody, antigen coating concentration, and substrate sensitivity were optimized using an indirect ELISA method described by Islam and Jones [41]. 

### 4.6. Determination of Specificity and Sensitivity of Inhibition ELISA

Tests that are developed for identification purposes should be able to discriminate between closely related and other venoms. In line with this, the specificity and sensitivity of the assay were determined according to the methods described by Sharma et al [24] and Armbruster and Pry [32]. The specificity of the assay was assessed by the observed percent inhibition at various sample antigen concentrations (6–0.008 µg/mL) in three homologous (*N. ashei*, *N. nigricollis*, *N. haje*) and two heterologous (*B. arietans and D. polylepsis*) venom samples (Table 1). From the three-fold serial dilution of the samples, the percent inhibition was calculated across all the inhibitor concentrations. Inhibition patterns were then compared between the homologous and heterologous samples. Similarly, to determine sensitivity of the inhibition ELISA, crude *N. ashei* venom was analyzed from an initial concentration of 27 µg/mL to a final concentration of 0.04 µg/mL following a three-fold serial dilution (Table 5). Samples were analyzed in duplicates. At the same time, 12 negative control samples that consisted of pre-immune mice serum samples and sample buffer were analyzed. The negative controls (devoid of the analyte of interest) were used for determining the limit of detection. The mean, standard deviation, and percent inhibition were then calculated. The limit of detection (LOD) of the assay was thus determined according to the method described by Armbruster and Pry [32] using the formula below based on the signal-noise approach;

LOD=concentration of antigen in well with % inhibition>mean+2×SD of negative controls

### 4.7. Inhibition ELISA for Detecting Three-Finger Toxins in Crude N. ashei Venom

The method described by Sharma and colleagues [24] with modifications was used. Wells of a 96-well maxisorp plate (NUNC, Denmark) were coated overnight at 4 °C with 100 µL of 1 µg/mL purified 3FTx toxin constituted in 0.1 M carbonate-bicarbonate buffer, pH 9.6. On the next day, 150 µL of the sample antigen (crude *N. ashei* venom) prepared to a final concentration of 27 µg/mL in blocking buffer (2% BSA + 0.05% PBST, pH 7.4) was added to row A of a separate plate (2). One hundred microliters (100 µL) of blocking buffer was added to rows B to H. A three-fold serial dilution was then performed by transferring 50 µL from row A to B and so on until G. The final 50 µL was discarded. Serum sample containing primary antibodies was prepared in blocking buffer at 1:1000 and 100 µL added to the duplicate wells of the same plate (including row H). The plate was then incubated at 37 °C for 60 min. The coating buffer in the earlier plate (1) was then aspirated and the plate washed three times with the washing/blocking buffer. The plate was then blocked with 200 µL of blocking buffer per well for 60 min at room temperature, following which washing was done as described previously. The content of the plate (2) containing the sample antigen and primary antibody was then transferred to the coated plate (1) and incubated at 37 °C for 60 min. The plate was washed again three times and 100 µL of goat-anti mouse IgG conjugated to horseradish peroxidase (American Qualex, San Clemente, CA, USA) added to each well. After a one-hour incubation at 37 °C, the washing step was repeated (with an extra wash) and 200 µL of 5 mg OPD substrate (Sigma Aldrich, St. Louis, MO, USA) added to the wells. The plate was again incubated for 60 mins at 37 °C for color development and then stopped with 50 µL per well of 3 M H_2_SO_4._ The plate was then read at 492 nm with a plate reader (Multiscan EX reader, Thermo Scientific, Massachusetts, MA, USA). Row H was considered the ‘No Antigen Control’ (NAC) and had its wells containing the blocking buffer and primary antibody without sample antigen. The NAC wells recorded the highest ODs. Subsequently, the percentage inhibition of the different wells containing different concentration of the sample antigen was determined against the absorbance of the NAC as per the formula below

$$\mathrm{Percent}\;\mathrm{inhibition}=\frac{\mathrm{NAC}\;\mathrm{OD}-\mathrm{Test}\;\mathrm{sample}}{\mathrm{NAC}\;\mathrm{OD}}\times 100$$

#### Testing Inhibition ELISA for Detecting 3FTxs in Other *Naja* sp. and Non-*Naja* sp. Venoms

The inhibition ELISA described above was tested for its ability to discriminate between venoms containing 3FTxs and those without using crude venoms from two other *Naja* sp. (*N. nigricollis* and *N. haje*) and two non-*Naja* sp. (*B. arietans* and *D. polylepsis*). The inhibition ELISA protocol described in Section 4.7 was implemented, except that the sample antigen concentration for all venoms was prepared to a final concentration of 6 µg/mL in the diluent previously described. Thus, a six point three-fold serial dilution was performed until a final concentration of 0.008 µg/mL sample antigen was obtained. All washing, blocking, and incubation steps were carried out as previously described. The plate was finally read at 492 nm and the percentage inhibition per well determined.

### 4.8. Detection of Three-Finger Toxins in Spiked Samples

The inhibition ELISA described earlier was also tested for its capacity to detect 3FTxs proteins in blood samples spiked with crude *N. ashei* venom. To spike samples, blood samples were collected from mice and then mixed with crude venom. Whereas the components of the assay were similar to the one described earlier, the sample collection and preparation were slightly different. In this experiment, six different Swiss mice were bled through the tail vein at different times—30 min, 1 h, 2 h, 6 h, 8 h, and 24 h. Immediately after sample collection, blood samples were spiked with equal volume of reconstituted crude *N*. *ashei* venom. Samples were then stored at −20 °C until assayed as earlier described. Pre-immune samples were added as negative control. The % inhibition was determined taking into account the NAC and the individual OD values.

### 4.9. Detection of Three-Finger Toxins in N. ashei Venom-Challenged Mice

The applicability of the inhibition ELISA in snakebite envenoming diagnostics was tested in mice challenged with crude *N. ashei* venom. Post the challenge, mice were bled in the tail vein at 30 min, 1 h, 2 h, 4 h, 6 h, 8 h, and 24 h. Pre-immune samples were added as negative control. The samples were processed, subjected to the inhibition ELISA procedure and % inhibition determined based on the OD values.

### 4.10. Data Analysis

Data analysis was done using GraphPad version 8.4.3 software (GraphPad Software, San Diego, CA, USA) and Microsoft Excel 2013. Differences in group means was determined by one-way ANOVA. In comparing the means within the comparisons, the one-way ANOVA was proceeded by Tukey’s or Dunnett’s post hoc multiple comparison test. At *p*-value < 0.05 cut-off, all comparisons were considered statistically significant.

## 5. Conclusions

The study reports an inhibition ELISA model and its potential application in snakebite envenoming, especially in relation to cobra bites. Albeit not for diagnostic purposes, the inhibition ELISA described herein demonstrates the possibility of detecting three-finger toxins present in the venoms of cobra snakes. Venoms of all five snakes tested with the assay are considered ‘category 1’, constituting the greatest threat to public health [42]. The detection of 3FTxs proteins in venom-challenged mice samples may suggest that the inhibition ELISA assay could potentially be used in detecting the presence or otherwise of the same in human clinical samples. However, the utility of this assay in human envenoming, thus has to be adequately evaluated. Also, studies involving a wide range of venoms may be necessary to further test the sensitivity of the assay.

## Figures and Tables

**Table 1:** Percent inhibition of 3FTxs induced by *N. ashei*, *N. nigricollis*, *N. haje*, *B. arietans*, and *D. polylepsis* venoms.

Inhibitor Concentration (µg/mL)	*Naja ashei*		*Naja nigricollis*		*Naja haje*		*Bitis arietans*		*Dendroapsis polylepsis*	
	OD	% Inhibition	OD	% Inhibition	OD	% Inhibition	OD	% Inhibition	OD	% Inhibition
6.000	0.094	76.90	0.085	77.68	0.088	79.75	0.474	7.06	0.458	8.77
2.000	0.118	71.13	0.087	77.01	0.091	79.17	0.464	9.02	0.439	12.46
0.667	0.174	57.25	0.098	74.24	0.098	77.45	0.454	11.08	0.458	8.67
0.222	0.259	36.49	0.125	66.97	0.113	73.99	0.467	8.53	0.465	7.28
0.074	0.295	27.64	0.177	53.37	0.131	69.85	0.471	7.65	0.445	11.27
0.025	0.319	21.62	0.252	33.42	0.182	58.11	0.447	12.45	0.434	13.46
0.008	0.331	18.80	0.299	21.00	0.260	40.28	0.447	12.35	0.424	15.55
NAC	0.407		0.379		0.435		0.510		0.502	

**Table 2:** Percent inhibition for determination of assay sensitivity.

% Inhibition for Determining Sensitivity of Inhibition ELISA				
	*N. ashei* Venom		Negative Controls (*n* = 12)	
Conc (µg/mL)	Replicate 1	Replicate 2		
27.00	76.69	78.68	49.60	49.70
9.00	75.28	77.22	47.71	48.91
3.00	69.62	76.04	46.52	45.97
1.00	64.39	70.54	26.80	20.83
0.33	54.18	56.96	25.09	25.82
0.11	45.02	44.95	20.46	21.92
0.04	34.44	38.55	-	-

**Table 3:** Inhibition of 3FTxs by sample antigen in crude *N. ashei* venom.

Inhibitor Concentration (µg/mL)	OD (492 nm)	% Inhibition
27.00	0.380	77.70
9.00	0.404	76.29
3.00	0.461	72.95
1.00	0.552	67.61
0.33	0.756	55.63
0.11	0.938	44.95
0.04	1.081	36.56
No antigen control (NAC)	1.704	-

**Table 4:** One-way ANOVA results ^a^.

F	19.14
*p* value	<0.0001
*p* value summary	****
Significant diff. among means (*p* < 0.05)?	Yes
R squared	0.7185

**^a^** ANOVA summary table showing whether or not inhibition induced by the sample-containing homologous (*N. ashei*, *N. nigricollis*, and *N. haje*) and heterologous (*B. arietans* and *D. polylepsis*) venom samples are statistically significant. At a *p*-value of <0.0001, the differences were found to be significantly different. This experiment was carried out to determine how specific the assay is for homologous samples, while being non-specific for heterologous samples.

**Table 5:** Multiple comparisons ^b^.

Tukey’s Multiple Comparisons Test	Adjusted *p* Value	Significant?
*N. ashei* vs. *N. nigricollis*	0.5409	No
*N. ashei* vs. *N. haje*	0.0649	No
*N. ashei* vs. *B. arietans*	0.0034	Yes
*N. ashei* vs. *D. polylepsis*	0.0051	Yes
*N. nigricollis* vs. *N. haje*	0.7312	No
*N. nigricollis* vs. *B. arietans*	<0.0001	Yes
*N. nigricollis* vs. *D. polylepsis*	<0.0001	Yes
*N. haje* vs. *B. arietans*	<0.0001	Yes
*N. haje* vs. *D. polylepsis*	<0.0001	Yes
*B. arietans* vs. *D. polylepsis*	0.9999	No

**^b^** Tukey posthoc multiple comparisons test was carried out to determine where the differences in sample-induced inhibition existed following one-way ANOVA analysis. Venom samples were compared against one another in order to determine if the differences were statistically significant or not.

## Data Availability

Not applicable.

## Sample Availability

Samples of the compounds are available from the authors.
